# Classification of Alzheimer’s Disease Leveraging Multi-task Machine Learning Analysis of Speech and Eye-Movement Data

**DOI:** 10.3389/fnhum.2021.716670

**Published:** 2021-09-20

**Authors:** Hyeju Jang, Thomas Soroski, Matteo Rizzo, Oswald Barral, Anuj Harisinghani, Sally Newton-Mason, Saffrin Granby, Thiago Monnerat Stutz da Cunha Vasco, Caitlin Lewis, Pavan Tutt, Giuseppe Carenini, Cristina Conati, Thalia S. Field

**Affiliations:** ^1^Department of Computer Science, University of British Columbia, Vancouver, BC, Canada; ^2^Vancouver Stroke Program and Division of Neurology, Faculty of Medicine, University of British Columbia, Vancouver, BC, Canada; ^3^Department of Statistics, University of British Columbia, Vancouver, BC, Canada

**Keywords:** Alzheimer’s disease, mild cognitive impairment, speech, language, eye-tracking, machine learning, multimodal

## Abstract

Alzheimer’s disease (AD) is a progressive neurodegenerative condition that results in impaired performance in multiple cognitive domains. Preclinical changes in eye movements and language can occur with the disease, and progress alongside worsening cognition. In this article, we present the results from a machine learning analysis of a novel multimodal dataset for AD classification. The cohort includes data from two novel tasks not previously assessed in classification models for AD (pupil fixation and description of a pleasant past experience), as well as two established tasks (picture description and paragraph reading). Our dataset includes language and eye movement data from 79 memory clinic patients with diagnoses of mild-moderate AD, mild cognitive impairment (MCI), or subjective memory complaints (SMC), and 83 older adult controls. The analysis of the individual novel tasks showed similar classification accuracy when compared to established tasks, demonstrating their discriminative ability for memory clinic patients. Fusing the multimodal data across tasks yielded the highest overall AUC of 0.83 ± 0.01, indicating that the data from novel tasks are complementary to established tasks.

## Introduction

Dementia affects approximately 47 million individuals globally and is considered to be one of the costliest diseases in developed countries (El-Hayek et al., 2019). Alzheimer’s disease (AD) is the most common cause of dementia, contributing to 60–80% of cases (Kumar and Tsao, 2019). Despite its cost and prevalence, there are still no disease-modifying treatments for AD.

Successful disease-modifying therapies for AD are most likely to be effective in individuals without advanced neurodegenerative changes (Sperling et al., 2014; Reiman et al., 2016). These individuals, as well as individuals with pre-clinical or very early stage disease, are of particular interest for disease-modifying drug trials for dementia, as preventing decline appears to be more promising than reversing it (Trempe and Lewis, 2018). Current evidence suggests that pre-clinical pathological hallmarks of AD are present years before overt clinical symptoms occur (Vickers et al., 2016) and that both dementia and cognitive impairment can often go undetected (Lang et al., 2017). To detect AD or early stage disease, targeted screening and reassessment are critical (Rasmussen and Langerman, 2019).

Current screening strategies for clinical trials targeting pre-clinical AD are inefficient and expensive. Up to 80% of potential participants fail the screening process, leading to trials spending upwards of $100,000 USD per enrolled participant (Kolata, 2018). A substantial portion of these costs is from expensive and invasive screening strategies including lumbar puncture, advanced imaging, genetic testing, or extensive neuropsychological testing (Watson et al., 2014). To accelerate preventative clinical trials and to address underdiagnosis in the community, there is a strong need for an efficient, accurate, cost-effective, and scalable screening tool for AD and its earlier stages which can include a proportion of individuals with Mild Cognitive Impairment (MCI), or Subjective Memory Complaints (SMC).

With the goal of developing a high-throughput and non-invasive screening tool, we present a machine learning analysis of a new multimodal eye-tracking and language dataset integrating two novel tasks: *pupil calibration* and *memory description*. In our pupil calibration task, participants are asked to fixate on a target for 10 s. This may allow us to better capture potential square-wave jerks (involuntary eye-movements that interrupt fixation) which are linked to AD (Nakamagoe et al., 2019) or other neurodegenerative processes. Our memory description task asks participants to describe a pleasant past experience. This enables participants to speak in a more open-ended way and may allow us to capture additional language patterns that may not be evident during picture description or reading tasks. Our dataset also includes language and eye-tracking data from participants completing the two tasks well-described in the literature, *picture description* and *reading*, alongside the novel tasks. Based on the dataset of these four tasks, we perform machine learning experiments to classify individuals into an AD/MCI/SMC group or control group, using expert clinician diagnoses as ground-truth labels. For our classification analysis, we perform experiments using individual tasks, as well as combining tasks.

## Related Work

In this section, we review similar work in the context of our research. We first describe previous work involving AD/MCI classification using two separate modalities: language and eye-tracking. Next, we introduce work on multimodal approaches that use synchronized language and eye-tracking data. Last, we discuss limitations of the previous work and reiterate our contributions in this current article.

### Language Analysis

Clinical studies have shown that changes in both speech and language are linked to AD pathology and that these changes progress with disease severity (Sajjadi et al., 2012; Rodríguez-Aranda et al., 2016). Ahmed et al. (2013) examined Cookie Theft picture description task speech from 15 individuals with autopsy-proven MCI or mild AD from the Oxford Project to Investigate Memory and Aging (OPTIMA), and 15 age- and education-matched healthy controls. By manually annotating speech, they found that semantic and lexical content, in addition to syntactic complexity, declined with disease progression (Ahmed et al., 2013). This process of manual transcription and annotation is time-consuming, and inspired subsequent investigations into an automatic classification of AD/MCI vs. controls using natural language processing (NLP).

**Figure 1:**
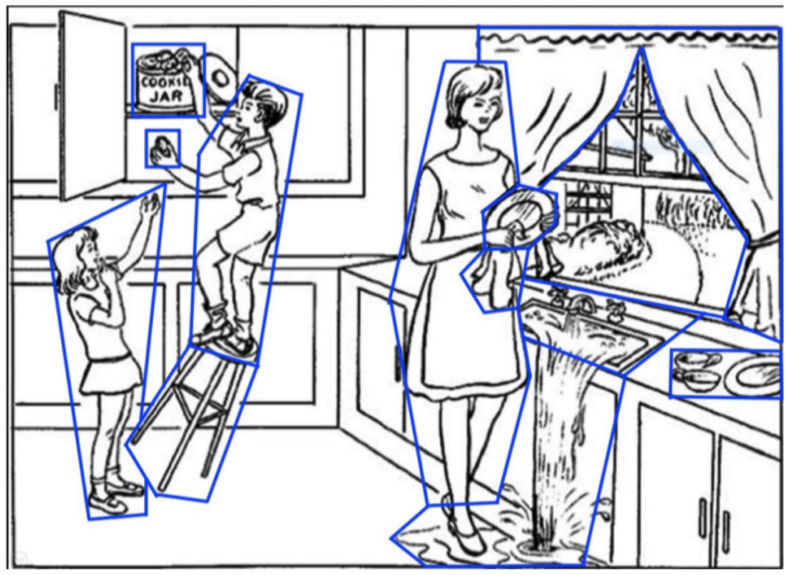
The picture description task (the Boston aphasia exam Cookie Theft picture). Areas of interest (AoIs) are shown as blue polygons.

Most research for AD/MCI classification using NLP has used data collected from the Cookie Theft picture description task—examples include the DementiaBank (Becker et al., 1994) and ADReSS datasets (Luz et al., 2020). DementiaBank is the largest publicly available dataset, containing picture description transcripts of 169 individuals with probable or possible AD, 19 with MCI, and 99 healthy controls (aged 45–90), collected between 1983 and 1988. In this task, participants are shown the Cookie Theft picture from the Boston Aphasia test (Figure 1) and are asked to describe everything they see (Goodglass and Edith, 1972). This task is commonly used for assessing spontaneous speech in AD and other clinical contexts (Cummings, 2019).

Traditional ML approaches have been used to classify AD/MCI vs. healthy controls through speech analysis (Orimaye et al., 2014; Fraser et al., 2016; Al-Hameed et al., 2017; Field et al., 2017; Masrani et al., 2017; Toth et al., 2018; Konig et al., 2018; Gosztolya et al., 2019). Orimaye et al. (2014) applied a variety of machine learning methods incorporating both lexical and syntactic features, to classify individuals in the DementiaBank dataset. This group compared different classifiers such as support vector machines (SVMs), naïve Bayes, decision trees, neural networks, and Bayesian networks. They found that SVMs showed the best performance, with the highest F-score of 74% (Orimaye et al., 2014). In a more recent study, Al-Hameed et al. (2017) used the DementiaBank dataset to extract acoustic features and built a regression model to predict cognitive assessment scores (Mini Mental State Exam scores, MMSE). Their model was able to predict MMSE scores with a mean absolute error of 3.1 using only acoustic features (Al-Hameed et al., 2017). Fraser et al. (2016) evaluated models incorporating a large variety of both linguistic and acoustic features from DementiaBank data. Using feature selection, they found that optimal classification performance was achieved when between 35 and 50 features were used. In a feature set of >50, performance dropped drastically. Overall, they achieved an accuracy of 81.96% in classifying individuals with AD from those without (Fraser et al., 2016). Building on this, our group improved accuracy to 84.4% (Field et al., 2017; Masrani, 2018) by adding features based on the clinical observation that hemispatial neglect occurs with AD (Drago et al., 2008).

More recently, deep learning approaches have further improved classification performance. Our group used a hierarchical attention Recurrent Neural Network (RNN) model incorporating both raw text and patient’s age, leading to 86.9% accuracy using DementiaBank data (Kong et al., 2019). Karlekar et al. (2018) achieved 91% accuracy using a Convoluted Neural Network (CNN)-RNN model trained on part-of-speech-tagged utterances. Using CNN on both DementiaBank and ADReSS data, Sarawgi et al. (2020) presented an ensemble of three models: disfluencies, acoustic, and intervention. Balagopalan et al. (2020) and Pappagari et al. (2020) showed that fine-tuned bidirectional encoder representations from transformers (BERT) outperformed models with hand-engineered features.

### Eye-Movement Analysis

AD alters ocular function. Neuronal death combined with neurofibrillary tangles and amyloid plaques in people with AD leads to cortico-cortical disconnections (Molitor et al., 2015) and infiltration of the locus ceruleus and associated projections (Granholm et al., 2017). These disconnection has been shown to primarily affect temporoparietal association areas, making individuals with AD more likely to develop visual, attentional, and eye movement disturbances (Garbutt et al., 2008) as well as pupillary dysfunction (Granholm et al., 2017). Examples of eye-movement disturbances in people with AD include abnormal saccadic behavior, saccadic intrusions, and slowed pupillary responses (Molitor et al., 2015).

These eye movement disturbances can be detected through a variety of tasks. For example, in reading tasks, AD patients have been shown to take longer to read text, have more fixations, re-read words more frequently, and are less likely to skip small and uninformative words (MacAskill and Anderson, 2016). Another study found that in a fixation task, saccadic gaze intrusions (such as square wave jerks) were associated with worsened cognitive test performance in people with AD (Bylsma et al., 1995).

Based on these results, eye movements have been investigated as another modality for automatic classification for AD/MCI, showing potential in a number of investigations. Pavisic et al. (2017) analyzed eye-movement data from 36 individuals with young onset AD, and 21 age-matched healthy controls. The participants completed three tasks in total: a fixation stability task (fixate on a point for 10 s without blinking), a pro-saccade task (looking at a target as soon as it appears), and smooth pursuit (following a moving target). The authors achieved the highest accuracy of 95% using hidden Markov models. Biondi et al. (2017) collected eye movement data from 69 participants with probable AD and 71 age-matched controls while they completed a sentence-reading task. This group reported the highest accuracy of 87.78% using an autoencoder approach that incorporates information derived from fixations, saccades, and sentence length from individuals.

### Multimodal Analysis

Recent multimodal work has demonstrated that language and eye movements act synergistically, further increasing AD classification accuracy. Fraser et al. (2019) had 26 participants with MCI and 29 healthy volunteers complete a paragraph-reading task and included additional speech-only data from the picture description task. Their best classification accuracy was 83% using a cascaded multimodal and multi-task classification approach incorporating comprehension question-related features, custom lexical and acoustic features, as well as eye-tracking features related to saccades and fixations. In our previous investigation, we analyzed multimodal data from 68 participants with SMC/MCI/AD and 73 controls completing the Cookie Theft picture description task. The best performance for language-only and eye-movement-only models was AUC of 0.73 and 0.77, respectively. A late fusion approach combining multimodal language and eye movement data significantly increased overall performance to 0.80 (Barral et al., 2020).

### Addressing the Gap in Literature

Our work is in line with previous work investigating language and eye tracking in combination for AD/MCI classification. However, ours is distinct from previous work in the following ways. First, while previous work has shown the discriminative ability of more constrained tasks (picture description and reading), these tasks may not be sufficient to capture the highly heterogeneous clinical manifestations of AD. Therefore, we designed and explored two additional tasks (pupil calibration and memory description) to capture key features linked to AD that may otherwise be missed with the established tasks. Second, while previous work focused mostly on extracting new features and optimizing single-task performance, we explored different tasks to determine if they can be used to increase overall performance. Third, compared to previous work that mostly focused on the DementiaBank corpus, which was gathered in the mid-1980s, our contemporary cohort incorporates current clinical practice for AD and MCI diagnosis, with a sample representative of current memory clinic populations and controls. Finally, while previous studies on contemporary datasets are limited by their small sizes (*n* ranging from 55–86), our cohort is larger (*n* = 162).

## Materials and Methods

In this section, we describe our cohort and provide a detailed description of the four tasks and how we collected data from the cohort for each task. We also explain data preprocessing, features and algorithms for classification, and our machine learning experiment settings.

### Data Collection

#### Cohort

Participants were recruited from a specialty memory clinic (“patients”) from a catchment area of 4 million (British Columbia, Canada), or from the community (“controls”), with efforts made to target recruitment to age- and sex-match patient participants. All participants were fluent in English, able to provide informed consent, could carry on a spontaneous conversation, and were aged 50 years or older. Clinic patients had a diagnosis of either SMC, MCI, or AD (mild or moderate stage). Patients were excluded if they had an active psychiatric disease, or any other neurological conditions apart from AD. Any participants with visual abnormalities or concerns that could impact eye tracking were noted. Diagnoses were made by expert clinicians using cognitive tests, neuroimaging, and laboratory data as per standard of care. The studies involving human participants were reviewed and approved by the University of British Columbia Clinical Research Ethics Board (Study ID# H17–02803). Participants provided their written informed consent.

Study data were collected and managed using REDCap electronic data capture tools hosted at the University of British Columbia (Harris et al., 2009, 2019). REDCap (Research Electronic Data Capture) is a secure, web-based software platform designed to support data capture for research studies, providing: (1) an intuitive interface for validated data capture; (2) audit trails for tracking data manipulation and export procedures; (3) automated export procedures for seamless data downloads to common statistical packages; and (4) procedures for data integration and interoperability with external sources. In our investigation, we used REDCap to capture all data outside of the language and eye-movement assessment, such as survey results and demographic information.

Recruitment is ongoing. The current cohort analyzed has 79 memory clinic patients (48 with mild to moderate AD, 22 with MCI, nine with SMC) and 83 healthy volunteers recruited between May 2019 and March 2020. The cohort characteristics including age, diagnosis, and MoCA scores are summarized in Table 1.

**Table 1:** Baseline demographic and clinical data.

		Patient	Control
Total participants	*N*	79	83
Age at enrollment	Average	72.09	65.63
	Range	53–96	50–92
	Standard deviation	9.1	9.8
Expert clinician diagnosis	Mild-moderate AD	48	
	MCI	22	
	SMC	9	
MoCA Score	Available scores (N)	75	83
	Average	20.0	27.3
	Range	3–30	19–30
	Standard deviation	6.2	2.6

#### Language and Eye-Movement Assessment

For each participant in our cohort, we collect language and eye movement data for the four tasks: pupil calibration, picture description, paragraph reading, and memory recall.

In the **pupil calibration task**, participants fixate on a static target for 10 s (Table 2). This fixation task aims to capture potential square-wave jerks characteristic of AD (Nakamagoe et al., 2019).

**Table 2:** Instructions and visual stimuli for each task.

Instructional prompt	Visual stimulus
**Pupil calibration task:** “A cross will appear in the middle of the screen. Please fixate your eyes on the cross. Do not look away from it. This will take about 10 s.”	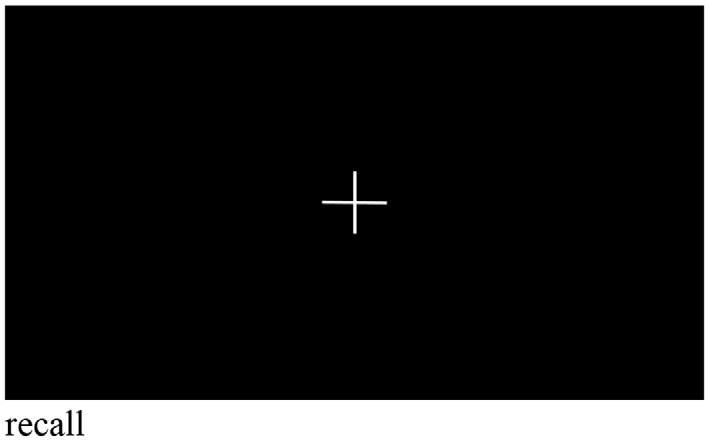
**Picture description task:** “You will be shown a picture on the screen. Describe everything you see going on in this picture. Try not to look away from the screen while describing the picture.”	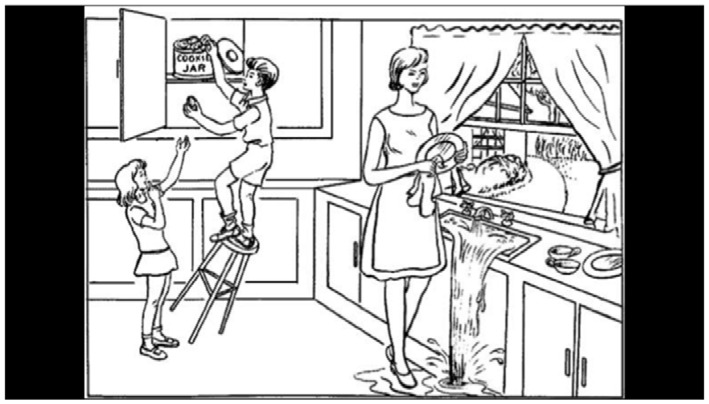
**Reading task:** “You will be shown a paragraph on the screen. Please read the paragraph out loud.”	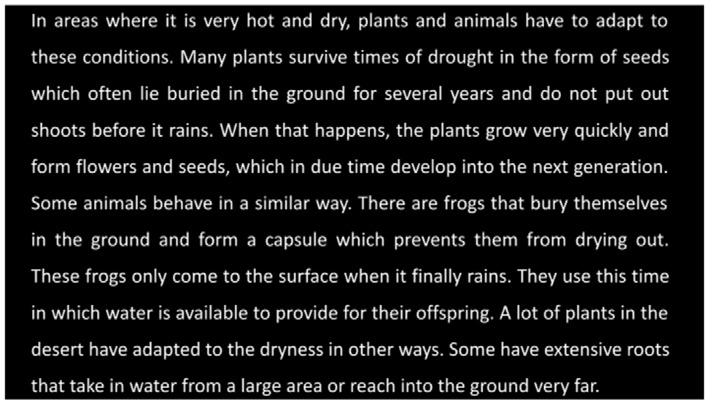
**Memory description task:** “Please recall a positive life event. Some examples are listed here: Your first job, how you met your best friend, a place you have traveled, your favorite teacher, your first pet, or the birth of your first child.”	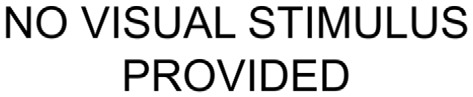

In the **picture description task**, participants describe the Boston Cookie Theft picture from the Boston Diagnostic Aphasia Examination (Goodglass and Edith, 1972; Table 2), a widely used and validated method for spontaneous speech assessment in a variety of clinical contexts, including Alzheimer’s disease (Cummings, 2019). It has been used in prior work for AD prediction using language (Fraser et al., 2016; Karlekar et al., 2018; Kong et al., 2019).

In the **reading task**, participants read a standardized paragraph aloud from the International Reading Speed Texts (IReST), an assessment tool for reading impairment designed to be readable at a sixth-grade level (Trauzettel-Klosinski and Dietz, 2012). The entire paragraph was presented to the participant at the same time to recreate a natural reading task, similar to a newspaper or book (Table 2). The goal of the reading task is to capture common reading-task deficits associated with AD, including reduced reading speed, and increased word fixations or re-fixations.

For the **memory description task**, participants describe a pleasant past experience to capture additional spontaneous speech data, with the goal of eliciting speech deficits that may be missed in a picture description or reading task. Additionally, the lack of visual stimuli (Table 2) allows the task to be completed identically despite possible variation in participant vision (e.g., low visual acuity, or blurred vision).

In summary, these tasks are designed around three dimensions—spontaneous vs. non-spontaneous speech, visual searching vs. fixation, as well as visual stimulus vs. no visual stimulus (Table 3). Owing to the variety of cognitive domains assessed through each of these dimensions, this may allow us to capture a broader range of AD/MCI-related discriminative language and eye movement data.

**Table 3:** Description of the three dimensions involved across the four tasks.

Task	Language	Visual activity	Visual stimulus
Pupil Calibration	*None*	Fixation	Yes
Picture Description	Spontaneous	Searching	Yes
Reading	Non-Spontaneous	Searching	Yes
Memory Description	Spontaneous	*None*	*None*

To assess baseline cognitive performance and to track potential risk factors for cognitive impairment, we administered a brief cognitive assessment, as well as a medical history and demographics questionnaire to each participant. Participants completed the Montreal Cognitive Assessment (MoCA), a 10-min pencil-and-paper cognitive screening test used by health professionals to assess MCI and AD (Nasreddine et al., 2005; Cordell et al., 2013). In addition, participants completed a demographic questionnaire and a medical history questionnaire. Participant responses were cross-checked against medical records (Supplementary Table 1).

For the language and eye movement battery, participants were seated at a testing platform, consisting of a monitor with a video/sound recorder and an infrared eyetracker affixed at the bottom of the monitor to record gaze and pupil size data. Participants were asked to keep looking at the screen during the battery and to avoid looking at the experimenter. Then, we calibrated the eye-tracking device by administering a standard 9-point eye-tracking calibration. Following successful calibration, participants performed the four tasks in order. Instructions and visual prompts for all tasks can be found in Table 2. All four tasks took approximately 10 min in total to complete.

Following the language and eye movement assessment, participants were asked to rate their experience with assessment by completing a 10-item questionnaire. Participants were asked to rate their comfort, interest, and willingness to repeat the assessment on a 4-point Likert scale, in order to determine the usefulness and scalability of the technology for routine assessment. We created and administered this questionnaire after recruiting the first 35 participants. As a result, only 127/162 participants completed this questionnaire (62 patients and 65 controls).

### Data Preprocessing

Following data collection, both gaze and speech recordings underwent pre-processing in order to standardize the format of the data and to facilitate subsequent experiments. Speech data was transcribed and timestamped either using Google Cloud speech-to-text service (*n* = 149), or manually (*n* = 13) if the participant requested to not have their voice data shared with the Google Cloud Platform. Following automatic transcription, human transcribers manually verified each transcript for accuracy. As the Google Cloud Platform does not transcribe filler words (e.g., “uh” or “um”) these were added manually by human transcribers. Additionally, as the Google Cloud Platform only transcribes utterances, unfilled pauses were manually marked as “[pause].” An unfilled pause was considered to be equal to or greater than 0.25 s of silence. The summary data statistics of the transcripts are in Table 4.

**Table 4:** Summary statistics of participant transcripts.

Task	Average # of sentences per transcript	Average # of words per sentence	# of words (total)	# of unique words (total)
Picture description	19.0	10.2	27,319	1,730
Reading	9.4	18.3	25,862	434
Memory description	17.8	11.9	28,801	3,026

To ensure data between modalities was aligned, timestamps of each transcript and gaze data file were manually checked against the screen recording of the visual stimulus. Manual transcribers corresponded timestamps of each task in the screen recordings to start and end timestamps in transcripts or gaze files. Additionally, task instructions were removed to include only participant speech in our analyses.

The Tobii Pro Studio software was used to export eye-tracking data. This includes fixations, saccades, and pupil size. Pupil size data was standardized by subtracting the mean pupil size during the pupil calibration task, described in “Language and Eye-Movement Assessment” section (Iqbal et al., 2005).

### Machine Learning Setup

#### Feature Engineering

After preprocessing the language and eye movement data, we then extracted linguistic and eye-tracking features from the raw data for AD/MCI classification.

##### Language Features

We used different sets of language features for the picture description, reading, and memory description tasks, respectively. Language features were not examined for the pupil calibration task as there was no speaking involved during this task. The entire feature set is summarized in Table 5.

**Table 5:** Abbreviated table of features used in the predictive models.

Task	Modality	Feature group and amount (*n*)
Pupil Calibration	Eye-Movement	Fixation (6), Saccade (22), and Pupil size (6).
Picture Description	Language	Cookie Theft image information units (13), Part-of-speech (15), Context-free-grammar rules (44), Syntactic complexity (24), Vocabulary richness (4), Psycholinguistic (5), Repetitiveness (5), and Acoustic (172).
	Eye-Movement	Fixation (6), Saccade (22), and Pupil size (6), Fixation on AoIs (9), Transitions to AoIs (2), and Pupil size when looking at AoIs (6).
Reading	Language	Syllable count (1), Pause count (1), Total duration (1), Total time spent speaking (1), Proportion of time spent speaking (1), Speech rate (1), Average syllable duration (1), Pauses per syllable (1), Pause rate (1), Pause duration (3), and Acoustic (172).
	Eye-Movement	Fixation (6), Saccade (22), and Pupil Size (6), Fixation on AoIs (9), Transitions to AoIs (2), Pupil Size when looking at AoIs (6), regression amplitude (3), regression distance (3), first-pass fixations (3), later-pass fixations (1), multi-fixations (1), re-fixation (1), reading fixation (1), re-reading fixation (1), and wrap-up gaze (3).
Memory Description	Language	Part-of-speech (15), Context-free-grammar rules (44), Syntactic complexity (24), Vocabulary richness (4), Psycholinguistic (5), Repetitiveness (5), and Acoustic (172).

*Details and heatmaps of the Top 10 features correlated with classification labels (patients vs. controls) are reported in Supplementary Table 2 and Supplementary Figures 1–4*.

For the picture description task, we extracted a comprehensive set of language features following (Fraser et al., 2016) as in our previous work (Field et al., 2017; Kong et al., 2019; Barral et al., 2020). These features comprise text features and acoustic features. The text features include part-of-speech, context-free-grammar rules, syntactic complexity, vocabulary richness, psycholinguistic, repetitiveness, and information units. Information unit features correspond to mentions of specific visual features in the picture description task. The acoustic features include Mel-frequency Cepstral Coefficients (MFCCs), which represent spectral information from speech signals transformed into the Mel-frequency scale (*n* = 172).

For the reading task, we use 12 task-specific features as in (Fraser et al., 2019). These features include syllable count, pause count, total duration, total time spent speaking, the proportion of time spent speaking, speech rate, average syllable duration, pauses per syllable, pause rate, and pause duration (max, mean, and standard deviation). These features aim to measure reading fluency such as speed, pauses, and disfluencies when reading, rather than assessing sentence formation or devising information unit features, as in the picture description task. This is due to the fact that all participants read the same paragraph, making spontaneous-speech-related features less meaningful.

For the memory description task, we extracted the same feature set as the picture description task, with the exception of information unit features. The information unit features were not used because while all other features aimed to assess basic language abilities such as fluency, syntax, or grammar, the information unit features are specific to the Cookie Theft picture.

For parsing and part-of-speech tagging, the Stanford CoreNLP was used. To obtain psycholinguistic features, the MRC database was used for concreteness, familiarity, and imageability of words. To detect pauses from audio, we used pydub, a Python package for audio processing.

#### Eye-Tracking Features

To capture participant eye movement and pupil behavior, we computed a set of summary statistics on fixations, saccades, and pupil size data. Fixations refer to a period of static gaze lasting 60 ms or longer, while saccades refer to quick movements between fixations. Pupil size refers to the actual physical pupil diameter of the pupil of each eye (as opposed to the perceived size depending on the view angle). The summary statistics for fixations and saccades include the sum, average, standard deviation, and max of the related gaze coordinates. In addition, we compute the count and rate for both fixations and saccades, as well as the distance, duration, speed, and angle for saccades only. For pupil size, we account for its average, standard deviation, and range. To compute these statistics, we used a similar approach as in related work (D’Mello et al., 2012; Lallé et al., 2016; Toker et al., 2017, 2019), which involves defining the duration of fixation, minimizing gaze location errors, and managing scanpath interruptions caused by blinking or head movement (Goldberg and Helfman, 2010). These rudimentary eye movement summary statistics for gaze features were used as features for all the tasks, except the memory description task. The entire feature set is summarized in Table 4.

For the pupil calibration task, we used only the eye-movement summary statistics for fixation, saccades, and pupil size as features, while participants stared at a fixed point on the screen.

For the picture description and reading tasks, which involve a complex visual stimulus with respect to pupil calibration (i.e., the picture to be described and text to be read by the participants), we defined additional features based on Areas of Interest (AoIs). An AoI is defined as any region of the input deemed relevant to the task. We use AoIs to bind gaze data to semantic information in the visual stimulus.

For the picture description task we defined Areas of Interest (AoIs) as features to encode elements in the Cookie Theft picture (Figure 1), As in our previous work (Barral et al., 2020), all AoIs used are analogous to information units (Croisile et al., 1996) from language features: *cookie, cookie jar, boy, girl, woman, stool, plate, dishcloth, water, window, curtain, dishes, and sink*. This was to capture important elements in the image that participants are likely to fixate on while completing the description task.

**Figure 2:**
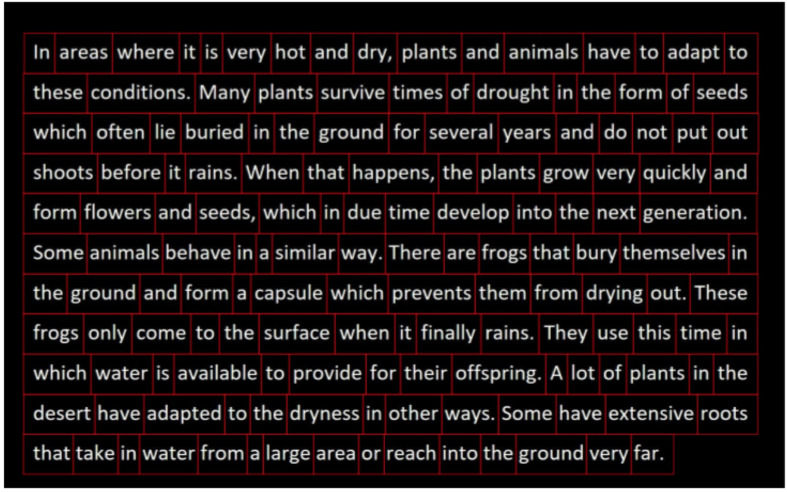
The reading task. AoIs are shown as individual red boxes.

For the reading task, we defined AoI features to encode each word in the paragraph reading task (Figure 2). The beginnings and ends of each sentence and line were marked as well. This was to correlate participant eye movements with progression through the reading task. We also incorporated reading-task-specific eye-tracking features from Fraser et al. (2019) such as fixation time for the last word in a sentence, the number of fixations on a word after the first pass, and the maximum number of words included in a regressive saccade. An abbreviated list of all feature groups can be found in Table 4.

For the memory description task, we also computed the same eye movement summary statistics as above. However, according to our preliminary analysis, all eye movement features from this task showed a poor correlation for AD/MCI classification. We speculate that this is due to the absence of visual stimulus during the task, causing participants to look at the screen randomly, or to look away from the screen towards the experimenter. For this reason, we have excluded eye movement data from the memory description task in further experiments.

We processed the eye tracking data using the Eye Movement Data Analysis Toolkit (EMDAT), an open source Python library. EMDAT produces a comprehensive set of eye tracking metrics specified over the entire display (task-agnostic), and over task-specific Areas of Interest (AoIs).

Due to difficulty calibrating the eye tracking device, or due to some participants having pre-existing eye conditions, we excluded 36 participants (19 patients and 17 controls) from subsequent gaze analysis in the pupil calibration, picture description, or reading tasks. These participants were either automatically rejected due to lack of samples by EMDAT, or manually rejected due to poor 9-point calibration results.

### Classification Strategies

We performed binary classification for patient and control groups. The patient group included individuals with AD, MCI, and SMC. We chose to analyze these heterogeneous diagnoses together within the “patient” category because our overall goal is to build a screening tool instead of a diagnostic tool. Further, in this way, we can identify highly predictive features shared across the entire disease spectrum.

To investigate the usefulness of our dataset, as well as the new and existing tasks for AD/MCI/SMC classification, we first evaluated single-task classification models using data from each of the four individual tasks and compared the performance of our novel tasks to the performance of the established tasks. Then, we assessed a task-fusion model to determine whether the new tasks can be used in combination with the established tasks to improve AD/MCI/SMC classification.

#### Classification With Individual Task Data

We first built our individual task models independently, testing for each of three different classification algorithms: Logistic Regression (LR), Random Forest (RF), and Gaussian Naïve Bayes (GNB). We selected these algorithms because they generated the best performances in our previous work using both eye-tracking and language collected from the picture description task (Barral et al., 2020). For tasks involving two modalities (language and eye movement, e.g., the reading task), we aggregated unimodal prediction probabilities using averaging, a widely used late fusion scheme (Battiti and Colla, 1994). In both (Fraser et al., 2019) and our previous work (Barral et al., 2020), late fusion outperformed early fusion for multimodal AD/MCI classification.

#### Classification With Combined Task Data

**Figure 3:**
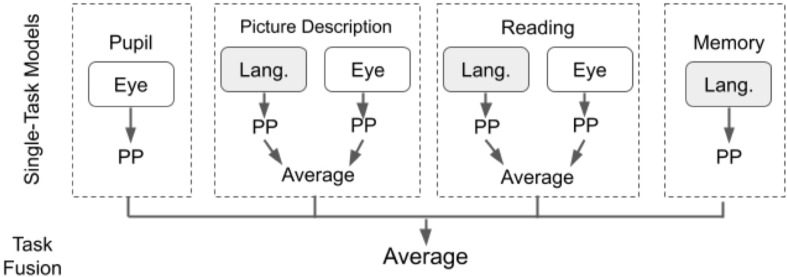
Diagram outlining the steps in modality and task fusion. Pupil, Pupil calibration; Memory, Memory description; PP, Prediction probability.

Next, we aggregated classification predictions across all tasks, with the goal of determining synergy between tasks. We present our task-fusion model in Figure 3, to combine predictions from all four tasks: pupil calibration, picture description, reading, and memory description. We first built individual task models using a single classification algorithm (as described in section Classification with Individual Task Data) and generated an output prediction for each task. Then, we used averaging to fuse results across all four task models. We report the results of task fusion for the three algorithms: LR, GNB, and RF. Note that each algorithm is used to process data for all tasks, in other words, we do not allow cross-algorithm task fusion.

For participants lacking eye movement data due to calibration problems (*n* = 36), we used only language data for classification, i.e., data from those participants were included only for learning individual task models using language.

#### Classification Settings

To perform classification, we used scikit-learn (v0.19.1), a Python package. We used default hyper-parameters in the scikit-learn APIs for each algorithm. For LR, we used L2 regularization. For GNB, we did not assign any prior probabilities of the classes. For RF, the number of trees was 10, the minimum number of samples required to split an internal node was two, and we allowed a tree to grow until all leaves were pure or until all leaves contained less than two samples. Gini impurity was computed for measuring the quality of a split.

To strengthen the stability of the results, we used stratified 10-fold cross-validation repeated 10 times on different stratified splits. Classification performance is reported in terms of Area Under the receiver operating characteristic Curve (AUC), calculated by averaging AUC results over the 10 folds and the 10 runs. We performed correlation feature selection (Hall, 1998) at each fold of cross-validation, to remove highly pairwise correlated features (Pearson *r* < 0.85) and features that showed very low correlation with the classification labels (patients vs. controls; *r* < 0.2). Note that we did not use feature selection for RF, as RF essentially performs feature selection as part of the algorithm.

### Statistical Analysis

To determine whether the two given model performances differ significantly from each other, we performed a series of statistical comparisons. In each comparison, we ran one-way ANOVA or two-way ANOVA tests with model AUC as the dependent variable. Classification models and tasks were used as factors. Following this, we completed subsequent pairwise *post hoc* testing *via* the Tukey Honestly Significant Difference (HSD), which adjusts for multiple comparisons.

Below we outline three alternate hypotheses to be tested statistically, as well as our testing methodology. A classifier was considered to be significant if the null hypothesis was rejected in each comparison.

1. **Novel tasks are significantly discriminative**. Here we compared the performance of novel task models to uniform dummy models trained on the same task data. The dummy model essentially classifies participants randomly (~0.5 AUC).
2. **Novel tasks are not significantly different from established tasks**. Here we compared the AUC of our novel task models to the AUC of established task models.
3. **Task fusion significantly outperforms individual tasks**. Here we compared the AUC of our task fusion models to the AUC of the individual task models.

### Feature Importance Analysis

To generate further insights from our classification experiments, we examined the most predictive features in our models. Since the LR model for task fusion was the best performing classifier, we investigated predictive features of each LR classification model (e.g., eye tracking classifier for the picture description task) used for the ensemble. Since LR models are linear, meaning the prediction is the weighted sum of the input values, we use the t-statistic for each coefficient to rank predictive features for classification.

## Results

In this section, we first outline the classification results from both individual tasks as well as the novel tasks and discuss the statistical significance of these results. Following this, we discuss the most predictive features determined by feature importance analysis. Finally, we present participant experience survey results.

### Classification Results

A summary of classification results is shown in Table 6. First of all, we found that both of our novel tasks, pupil calibration, and memory description, achieved reasonable classification accuracies of AUC 0.71 ± 0.01 and 0.78 ± 0.01, respectively. These significantly outperformed dummy classifiers (*p* ≤ 0.001), which suggests that these new tasks are discriminative (**H1**).

**Table 6:** Fusion model results compared to individual task model results, reported in AUC ± standard deviation.

Feature Set	Modality	N	GNB	LR	RF
Pupil Calibration (novel task)	Eye	126	**0.71 ± 0.02**	0.68 ± 0.02	0.63 ± 0.05
Picture Description	Eye	126	0.71 ± 0.02	0.73 ± 0.03	0.64 ± 0.04
	Lang	162	0.78 ± 0.01	0.77 ± 0.02	0.74 ± 0.02
	Eye + Lang	162	**0.80 ± 0.02**	0.79 ± 0.01	0.77 ± 0.02
Reading	Eye	126	0.70 ± 0.02	0.73 ± 0.02	0.72 ± 0.03
	Lang	162	0.79 ± 0.01	0.78 ± 0.01	0.78 ± 0.03
	Eye + Lang	162	0.78 ± 0.01	0.80 ± 0.01	**0.82 ± 0.02**
Memory (novel task)	Lang	162	**0.78 ± 0.01**	0.72 ± 0.02	0.72 ± 0.04
Task Fusion	Eye + Lang	162	0.82 ± 0.01	**0.83 ± 0.01**	**0.83 ± 0.02**

*The highest classification performance for each task is in bold. Mod, modality; Eye, eye-movement alone; Lang, language alone; Eye + Lang, eye-movement and language aggregate model. More evaluation metrics such as specificity and sensitivity are reported in Supplementary Table 3. The data in gray background represent unimodal model results when multimodal data were available. So, they were not used for our statistical analysis when we compared task models*.

Second, we performed comparisons between novel tasks models and established task models. We found that the novel task results are lower than established task results (language+eye-movement; *p* ≤ 0.001) with the exception of the memory task GNB model (*p* > 0.1). However, we did not find a significant difference when comparing language-alone models for the established tasks against the best memory task model (*p* > 0.1), with the exception of the picture description RF model, which was significantly outperformed by the memory GNB model (*p* ≤ 0.001). This trend was similar for eye-movement, which also showed no significant difference when comparing eye-movement-alone models for the established tasks against the best pupil calibration task model (*p* > 0.06). This indicates the novel tasks are performing similarly to the established tasks, with regard to their respective modality (**H2**).

Third, when we compared task-fusion to individual-task models, LR task-fusion models significantly outperformed all individual-task models (*p* ≤ 0.03). The other two task-fusion models were significantly better when compared to individual-task model performance, with a few exceptions (e.g., GNB task-fusion vs. GNB picture description, *p* > 0.37). This suggests that task-fusion has a synergistic effect, increasing performance over individual tasks (**H3**).

### Highly Predictive Features

Following classification experiments, we performed feature importance analysis. These results are found in Table 7.

**Table 7:** Top-ranked important features from the logistic regression models for each task and modality, with corresponding odds ratio and 95% confidence intervals.

Task	Modality	Feature	Direction	Odds ratio	95% CI
Pupil calibration	Eye movement	Standard deviation of saccade speed	+	1.46	(0.63, 3.37)
		Mean fixation duration	–	0.9997	(0.9989, 1.0005)
		Standard deviation of saccade distance	+	1.00	(0.99, 1.02)
		Ratio of time spent fixating to saccading	–	1.00	(0.98, 1.01)
		Sum of saccade distance	+	1.0003	(0.9988, 1.0019)
Picture description	Eye movement	Longest fixation on window AoI	–	0.998	(0.996, 1.000)
		Number of transitions from curtain AoI to window AoI	–	0.83	(0.66, 1.07)
		Number of transitions from boy AoI to cookie AoI	–	0.40	(0.10, 1.70)
		Time before first fixation on water AoI	+	1.00006	(0.99996, 1.0015)
		Number of transitions from dishcloth AoI to window AoI	+	2.57	(0.46, 14.52)
	Language	Mentions of exterior information unit	–	0.43*	(0.18, 0.99)
		Variance of acoustic feature (MFCC 8)	–	0.97	(0.93, 1.00)
		Distribution of acoustic feature (MFCC 6)	–	0.36	(0.11, 1.15)
		Mean of acoustic feature (MFCC 5)	+	1.17	(0.97, 1.41)
		Mean of acoustic feature (MFCC 4)	+	1.15	(0.95, 1.39)
Reading	Eye movement	Refixation count	+	1.03	(0.99, 1.09)
		Later pass first fixation count	+	1.04	(0.97, 1.11)
		Mean saccade distance	–	0.66	(0.25, 1.79)
		Mean wrap-up gaze duration	–	0.99	(0.98, 1.01)
		Fixation count	–	0.99	(0.96, 1.02)
	Language	Variance of acoustic feature (MFCC 12)	–	0.95*	(0.92, 0.99)
		Distribution of acoustic feature (energy acceleration)	+	1.17	(0.99, 1.38)
		Mean of acoustic feature (MFCC 3)	+	1.12	(0.99, 1.26)
		Variance of acoustic feature (MFCC 2)	–	0.99	(0.98, 1.00)
		Overall task duration	+	1.00004	(0.99999, 1.00010)
Memory description	Language	Variance of acoustic feature (MFCC 8)	–	0.98*	(0.95, 1.00)
		Variance of acoustic feature (MFCC 2)	–	0.99	(0.97, 1.00)
		Mean of acoustic feature (energy)	+	1.47	(0.80, 2.72)
		Distribution of acoustic feature (MFCC 4)	+	1.45	(0.75, 2.78)
		Variance of acoustic feature (MFCC 3)	+	1.01	(0.99, 1.02)

A positive (+) direction (odds ratio > 1) represents a higher feature value in the patient group. A negative (–) direction represents a higher feature value for the control group. *p = 0.05. Details of these features are reported in Supplementary Table 4.

In the pupil calibration task, we found that patients showed more variation in their eye movements, and had more eye movements overall. More variation is indicated by a higher standard deviation for saccade speed and distance compared to the control group. More eye movements are indicated by a lower mean fixation duration in patients compared to controls. As the task is only 10 s in total, a lower mean of fixations corresponds to shorter average fixations. This suggests that patients are more prone to refixation during the task.

In the eye-tracking model for the picture description task, three window-related AoIs were ranked as highly important: longest fixation on the window AoI, number of transitions from curtain AoI to window AoI, and number of transitions from dishcloth AoI to window AoI. This is in line with our previous work, where we found that four of the 10 top eye-movement features in the picture description task were related to the window AoI (Barral et al., 2020). This is especially noteworthy because the exterior information unit was ranked first overall among language features, and was the only text feature among top ranked features.

In general, acoustic features were generally ranked as more important than other language features in all three language-based tasks with two exceptions: information unit mentions (exterior), and duration of reading task. We found a higher task duration in the patient group for the reading task, suggesting that patients are more likely to take longer to complete the reading task.

### Results From Experience Questionnaire

The full results from the experience with the technology questionnaire can be found in Table 8. Most participants (>90%) answered “Agree” or “Strongly Agree” when asked if they felt comfortable, relaxed, engaged, or interested during the assessment. Few participants (11% of patients and 6% of controls) reported discomfort during the assessment. Very few participants (5%) reported having privacy concerns with the technology.

**Table 8:** (%) represents the proportion of patients or controls who answered “Agree” or “Strongly Agree” when asked each question.

Total responded (N)	Patient 62	Control 62
I experienced discomfort during the assessment	11%	6%
I was comfortable during the assessment	94%	95%
I was relaxed during the assessment	92%	97%
I have privacy concerns using this technology	5%	5%
I would be willing to do the assessment again	95%	100%
In a clinical setting: I would be willing to do the assessment once a year	94%	97%
I would be willing to do the assessment once a month	40%	51%
I would be willing to do the assessment once a week	16%	20%
I would be willing to do the assessment once a day	2%	9%
I was engaged and interested during the assessment	94%	98%

The majority of participants (>90%) reported their willingness to repeat the assessment again or to repeat the assessment on a yearly basis in a clinical setting. However, only some participants (40% of patients and 51% controls) reported that they were willing to repeat the assessment once a month. Even fewer participants (16% of patients and 20% controls) were amenable for weekly re-assessment, and less again (2% of patients and 9% controls) for daily re-assessment.

## Discussion

In this article, we present a new, contemporary, multimodal dataset for AD classification that includes two novel tasks and two established tasks. Importantly, our cohort is also considerably larger than other similar contemporary datasets.

This article is a substantial extension of a conference article previously published by our group (Barral et al., 2020), which showed the potential of eye movement data in combination with language data collected during the picture description task. Building on our previous work, we added three more tasks to explore the two data modalities, which include two completely novel tasks, pupil calibration, and memory description, as well as paragraph reading for AD/MCI/SMC classification. Second, the cohort in our current article has increased in size since the conference article (*n* = 162 vs. 141). Using multimodal data collected from new tasks, we aimed to assess the discriminative ability of these novel tasks for AD classification. We also aimed to confirm our previous finding that eye-tracking and language data increases classification performance, in a larger dataset with more tasks.

To our knowledge, this is the first investigation of pupil calibration and memory description tasks for AD/MCI/SMC classification. Our approach has several advantages over similar investigations. Here we build a high-quality dataset of synchronized speech and gaze data collected during four distinct tasks (two novel and two that have been studied more extensively in the field). Our cohort (*n* = 162) is also larger than other contemporary datasets which have included between 55 and 86 participants in total (Biondi et al., 2017; Pavisic et al., 2017; Toth et al., 2018; Fraser et al., 2019). Furthermore, our dataset incorporates current AD/MCI/SMC diagnostic practices, in contrast to the large DementiaBank cohort of picture descriptions for AD classification (Becker et al., 1994), which was collected in the mid-1980s and incorporates clinical diagnoses from best practices at that time.

### Discussion on Classification Results

Our analyses show that the novel tasks alone and in combination with previous tasks significantly outperform a dummy model, demonstrating their discriminative ability for AD/MCI/SMC. This suggests that eye movements collected during a fixation task and language data collected during an open-ended spontaneous speech task are discriminative of AD/MCI/SMC vs. controls.

Additionally, our results show that the established tasks outperform novel tasks, reinforcing and validating their continued use for classification. However, this variation in performance could also be attributed to the fact that our novel tasks only incorporate data from a single modality, either eye tracking or speech, as opposed to the multimodal established tasks. When comparing eye-movement-only model results, we found that the pupil calibration, picture description, and reading tasks all showed similar performance. This suggests that our novel pupil calibration task achieves similar performance to the established tasks when comparing the same modality. Similarly, when comparing language-only model results, we found that our novel memory task models had comparable performance to reading and picture description task models.

We also show that our best-performing task-fusion model (LR) significantly outperforms all individual-task models. This suggests that data from the four tasks in our assessment act synergistically to significantly improve the overall AD/MCI/SMC classification performance.

We found in both picture description and reading that fusing modalities improves performance over individual modalities. This observation reinforces what we found in our previous investigation for the picture description task (Barral et al., 2020). Additionally, these results in the reading task validate results from other investigations (Fraser et al., 2019).

### Task Dimension Comparison

When examining the dimensions of our tasks (Table 3) we first compared the performance of a non-spontaneous speech task (reading) to spontaneous speech tasks (picture and memory description). It was noteworthy that language-alone performance among all three of these tasks was similar considering that in the reading task all participants read the same paragraph, limiting word choices used in the task, as opposed to open-ended and spontaneous speech from the picture and memory description tasks. This suggests that speech characteristics captured by acoustic analysis (such as pause and speed) may be more discriminative in this investigation.

Second, we compared the results of searching (picture description and reading) vs. fixation (pupil calibration) tasks. Here, the eye-movement-only performance among all three tasks was found to be similar. The pupil calibration task yielded analogous results (best AUC of 0.71) to eye-movement models from the other tasks, especially considering its simplicity and short 10-s duration. This suggests that the pupil calibration task could be a very good candidate for high-throughput screening.

Not only does the pupil calibration task perform well, our feature importance results suggest that the task classifier may also be capable of capturing abnormal saccadic behavior associated with AD. In particular, we observed that the most discriminative features in this task showed that patients tended to have shorter fixations, more eye movements, and more varied eye movements during the task. This abnormal saccadic eye movement behavior may be attributed to AD-related amyloid plaques in the brainstem (Parvizi et al., 2001), affecting premotor burst neurons responsible for generating saccades (Scudder et al., 2002; Otero-Millan et al., 2011). Such results make the pupil calibration task highly promising for future investigations, especially as it is brief, efficient, and could be readily implemented and measured using existing webcam technology.

Third, we compared the results of tasks with visual stimuli to tasks with no visual stimuli (memory description). Our models trained on eye movement data collected during the memory description task had essentially random results. This suggests that tasks that aim to collect eye movement data should include a visual stimulus.

### Discussion on Experience Questionnaire

We also show that our four-task assessment is highly tolerable in our target population. The large majority (>90%) of older adult controls and patients reported that they were comfortable and relaxed during the assessment. The majority of our target population also did not have privacy concerns with recording and analyzing speech, video, or eye-tracking for classification. This suggests that our assessment would be appropriately applied to this population for the purpose of non-invasive AD/MCI screening. Targeted screening of older adults is key for detecting AD/MCI in the community (Rasmussen and Langerman, 2019), and it is important that this is as tolerable as possible for participants. Screening can also help identify AD/MCI early, which improves long-term prognosis in cognitively impaired individuals (Rasmussen and Langerman, 2019).

While baseline screening is important, follow-up screening is also key to detecting longitudinal changes in cognition in keeping with disease progression (Rasmussen and Langerman, 2019). Our findings also show that our assessment would be suitable for re-screening, with the majority of participants reporting that they are amenable to annual re-assessment.

### Limitations and Future Work

**Size of the dataset**: A key limitation of our work is the size of our dataset. Despite our dataset being larger than other contemporary AD/MCI classification datasets (Biondi et al., 2017; Pavisic et al., 2017; Toth et al., 2018; Fraser et al., 2019), more advanced machine learning algorithms such as deep learning-based methods are powered by large datasets. Even the traditional machine learning approaches used in this investigation would benefit from a larger dataset. Recruitment and follow-up are ongoing, with a goal of 500 participants overall (250 patients and 250 controls). With a larger dataset, we aim to explore more sophisticated machine learning models, more advanced feature selection, and additional task fusion schemes.

**Accessibility of eye-tracking device**: One possible limitation could be the resolution and quality of our eye-tracking device. More sensitive eye-tracking devices can track microsaccades, which may allow for better discrimination between patients and controls. However, these devices require head fixation with a chin rest and forehead strap and with more sensitive and time-consuming calibration. Thus may be unsuitable or uncomfortable for older adults, particularly with degenerative cervical spine changes. To maximize participant comfort, we instead chose to use the current eye-tracking device (Tobii Pro X3-120), as this allowed for eye-movement data to be collected while the participant is comfortably seated in a regular chair without head fixation. In the future, more comfortable eye-tracking technology with better resolution may become available, or eye data collection based on webcam and phone camera recordings may become more feasible. This would allow scalable and remote assessment which could also be integrated into mobile devices.

**Possible misclassification of control subjects or patients**: Our participants are recruited from a memory clinic, based on expert clinician diagnoses made using test scores, neuroimaging, as well as laboratory or genetic data. Although our control subjects did not carry a diagnosis of neurodegenerative disease, without detailed phenotyping with detailed imaging, laboratory, and clinical assessment, we cannot exclude the possibility that some control subjects may have an undiagnosed mild cognitive impairment, Alzheimer’s disease, or other pathology that could contribute to misclassification.

Furthermore, our use of expert clinician diagnoses for our patients may also be a source of mislabelling. One large post-mortem study found that AD diagnostic sensitivity and specificity can be as low as 70.9% and 44.3%, respectively (Beach et al., 2012), and approximately 30% of people with MCI that develop dementia do not meet the pathological criteria for AD (Jicha et al., 2006). This limitation is not unique to this investigation, with several dementia trials using expert diagnosis as an inclusion criterion (Cummings et al., 2020).

**Younger control group**: Despite efforts for targeted recruitment to age-match both cohorts, the average age of our patient group (72.09 ± 9.1) remains higher than the average age of our control group (65.63 ± 9.8). As a result, it is possible that some of the differences between patients and controls may be attributed to differences in speech or gaze related to normal aging. For example, the control of eye movements and speech can be impacted by both healthy aging-related and AD-related neurodegeneration in the cerebral regions, spanning brainstem to neo-cortex (Murphy et al., 1997; Pierrot-Deseilligny et al., 2004), and could have impacted certain speech-gaze features, such as repeat word mentions or visual re-fixations. We plan to explore age-related task differences in our control group as a future direction.

**Multimodal features**: In our feature importance analysis, we found that features related to the window- or the exterior of the home in the Cookie theft photo were ranked highly in both eye-tracking and language task models. This finding suggests an interesting future direction using multimodal features leveraging both eye-tracking and language simultaneously e.g., time delays between fixating on an AoI, and saying the related information unit. We plan to explore multimodal features in the future to capture potential deficits in coordinated eye movement and language.

**Classification vs. risk-stratification**: In this work, we aimed to build upon work for classifying individuals with established AD/MCI/SMC in a cross-sectional cohort. In the future, we aim to create a tool that could also risk-stratify for progression of neurodegenerative disease (i.e., progressing from SMC to MCI, MCI to AD, or from early-stage AD to later AD stages). To this end, we are performing longitudinal reassessments every 6 months up to 24 months for future risk-stratification models.

## Conclusion

Our results show that our multimodal screening assessment is well-tolerated and discriminates between memory clinic patients and healthy controls. We also show that our novel tasks can be leveraged in combination with established tasks to bolster overall AD/MCI/SMC classification with task fusion. These results are highly promising for future investigations into non-invasive and automatic AD/MCI/SMC classification.

## Data Availability Statement

The datasets presented in this article are not readily available because study participants have not consented to the distribution of their personally identifying data (such as audio and video recordings). Requests to access the datasets should be directed to TF, http://thalia.field@ubc.ca.

## Ethics Statement

The studies involving human participants were reviewed and approved by University of British Columbia Clinical Research Ethics Board (Study ID# H17-02803). The patients/participants provided their written informed consent to participate in this study.

## Author Contributions

GC, CC, and TF contributed to the conception, design, and oversight of the study. TS, SN-M, CL, and PT recruited study participants and administered study assessments. HJ, OB, AH, and SG designed machine learning features and performed all machine learning experiments. TS organized the database. MR and TC performed the statistical analysis. TS and HJ wrote the first draft of the manuscript. MR, AH, SN-M, CL, PT, and TC contributed to sections of the manuscript. All authors contributed to the article and approved the submitted version.

## Conflict of Interest

The authors declare that the research was conducted in the absence of any commercial or financial relationships that could be construed as a potential conflict of interest.

## Publisher’s Note

All claims expressed in this article are solely those of the authors and do not necessarily represent those of their affiliated organizations, or those of the publisher, the editors and the reviewers. Any product that may be evaluated in this article, or claim that may be made by its manufacturer, is not guaranteed or endorsed by the publisher.
