# Supplementary material for: Classification of Alzheimer’s Disease Leveraging Multi-task Machine Learning Analysis of Speech and Eye-Movement Data
Source: Front Hum Neurosci. 2021 Sep 20;15:716670. doi: 10.3389/fnhum.2021.716670 (PMC8488259; doi:10.3389/fnhum.2021.716670)
Supplement: Supplementary file 1 [file Data_Sheet_1.docx]

**Supplementary Material**

Supplementary Table 1. Full results from cohort demographic, diagnosis, medical history, and cognitive test data.

|  |  | **Patient** | **Control** |
| --- | --- | --- | --- |
| Total Participants | N | 79 | 83 |
| Sex | Male | 40 | 59 |
|  | Female | 39 | 24 |
| Expert clinician diagnosis | Mild-Moderate AD | 48 |  |
|  | MCI | 22 |  |
|  | SMC | 9 |  |
| Age at enrollment | Average | 72.09 | 65.63 |
|  | Range | 53-96 | 50-92 |
|  | Standard deviation | 9.12 | 9.77 |
| Years of education | Average | 14.8 | 15.0 |
|  | Range | 10-21 | 10-18 |
|  | Standard deviation | 2.8 | 2.3 |
| Medical history (N) | Previous stroke | 8 | 2 |
|  | Parkinson’s disease | 0 | 0 |
|  | Other neurological diagnosis | 1 | 3 |
|  | History of major depression | 7 | 10 |
|  | Other psychiatric condition | 6 | 9 |
|  | Previous concussion | 21 | 19 |
|  | Hypertension | 31 | 24 |
|  | Hyperlipidemia | 19 | 18 |
|  | Epilepsy | 1 | 1 |
|  | HIV | 1 | 1 |
|  | REM sleep disorder | 2 | 0 |
|  | Sleep impaired | 24 | 31 |
|  | Family history of dementia | 43 | 37 |
| Functional independence (N) | Instrumentally independent (IADL) | 58 | 83 |
|  | Functionally independent (ADL) | 71 | 83 |
|  | Functionally dependent (ADL) | 8 | 0 |
| History of substance use | Currently smoking | 4 | 8 |
|  | Former smoker | 26 | 30 |
|  | ≥ 4 alcoholic drinks per week | 17 | 19 |
|  | 2-4 alcoholic drinks per week | 19 | 18 |
|  | ≤ 2 alcoholic drinks per week | 17 | 18 |
|  | Never drinks alcohol | 26 | 18 |
|  | Previous substance use | 9 | 11 |
| Sleep routine | Average hours of sleep per night | 7.6 | 7.0 |
|  | Range | 3-12 | 3-11 |
|  | Standard deviation | 1.5 | 1.3 |
| MoCA score | Missing scores (N) | 4 | 0 |
|  | Mean | 20.0 | 27.3 |
|  | Range | 3-30 | 19-30 |
|  | Standard deviation | 6.20 | 2.56 |

Supplementary Table 2. Details and descriptive statistics about the top 10 features most correlated with the classification labels (patients vs. controls). All features used are continuous. In the unit column “-” means the unit is not human intuitive.

| **Task** | **Modality** | **Feature** | **Unit** | **Max** | **Mean** | **Median** | **Min** | **Standard deviation** |
| --- | --- | --- | --- | --- | --- | --- | --- | --- |
| Pupil | Eye movement | Standard deviation of saccade speed | degree/s | 15 | 1.2 | 0.9 | 0 | 1.4 |
|  |  | Standard deviation of saccade distance | degrees | 361.7 | 75 | 56.8 | 0 | 69.8 |
|  |  | Mean saccade distance | degrees | 409.8 | 95.6 | 79.7 | 0 | 62.6 |
|  |  | Sum of saccade distance | degrees | 5834.5 | 841.8 | 632.8 | 33.1 | 807.9 |
|  |  | Fixation count | count | 52 | 19 | 19 | 1 | 12 |
|  |  | Sum of path distance | degrees | 1883.8 | 546.8 | 522.4 | 2.2 | 403.3 |
|  |  | Minimum pupil size | mm | -0.1 | -0.2 | -0.2 | -0.5 | 0.1 |
|  |  | Mean of saccade duration | ms | 55.3 | 29.4 | 27.7 | 0 | 9 |
|  |  | Mean of fixation duration | ms | 8274 | 855.2 | 469.6 | 101.1 | 1077.9 |
|  |  | Ratio of time spent fixating to saccading | - | 367.1 | 55.6 | 38.8 | 3.5 | 53.1 |
| Picture Description | Eye movement | Time before first fixation on "water" AoI | ms | 59877 | 11786.7 | 9961 | 1367 | 10378.5 |
|  |  | Longest fixation on "window" AoI | ms | 2391 | 649.9 | 571 | 175 | 341.4 |
|  |  | Number of transitions to "boy" AoI from "woman" AoI | count | 0.0909 | 0.014 | 0.011 | 0 | 0.017 |
|  |  | Time before first fixation on "sink" AoI | ms | 82891 | 10178.1 | 7812 | 800 | 10587.4 |
|  |  | Number of transitions from "cookie" AoI to "girl" AoI | count | 4 | 0.44 | 0 | 0 | 0.77 |
|  |  | Mean fixation duration on "stool" AoI | ms | 650 | 195.3 | 185 | 67 | 85.1 |
|  |  | Number of transitions to "window" AoI from "woman" AoI | count | 0.1 | 0.0132 | 0.0079 | 0 | 0.0158 |
|  |  | Number of transitions to "girl" AoI from "jar" AoI | count | 0.125 | 0.0131 | 0.0076 | 0 | 0.0185 |
|  |  | Fixation rate on "water" AoI | - | 0.0127 | .00650 | 0.00619 | 0 | 0.00248 |
|  |  | Longest fixation on "water" AoI | ms | 1425 | 340.7 | 300 | 83 | 218.3 |
|  | Language | Skewness of acoustic feature (energy) | - | 2.4 | 0.4 | 0.3 | -0.7 | 0.5 |
|  |  | Variance of acoustic feature (MFCC 8) | - | 189.8 | 120.5 | 120.6 | 61.6 | 28.8 |
|  |  | Variance of acoustic feature (MFCC 1) | - | 252 | 128.3 | 127.3 | 27.1 | 44 |
|  |  | Variance of acoustic feature (MFCC 2) | - | 300.4 | 124.7 | 119.2 | 30.4 | 48.7 |
|  |  | Variance of acoustic feature (MFCC 5) | - | 296.8 | 137.6 | 139.3 | 56.1 | 41.2 |
|  |  | Kurtosis of acoustic feature (MFCC 2) | - | 5.4 | 0.5 | 0.3 | -0.8 | 1.1 |
|  |  | Kurtosis of acoustic feature (MFCC 7) | - | 3.4 | 0.7 | 0.6 | -0.4 | 0.7 |
|  |  | Kurtosis of acoustic feature (MFCC 1) | - | 10.4 | 2 | 1.5 | -0.1 | 1.6 |
|  |  | Kurtosis of acoustic feature (MFCC 8) | - | 5.8 | 1.2 | 1.1 | -0.3 | 0.9 |
|  |  | Kurtosis of acoustic feature (MFCC 5) | - | 4.9 | 0.9 | 0.8 | -0.5 | 0.9 |
| Reading | Eye movement | Later pass first fixation count | count | 131 | 36.6 | 32 | 2 | 22 |
|  |  | Mean of scanpath angles between saccades and horizontal axis | radiants | 1.4 | 1 | 1 | 0.6 | 0.2 |
|  |  | First pass first fixation count | count | 140 | 73.5 | 77 | 3 | 29.7 |
|  |  | Ratio of scanpath angles between consecutive saccades | - | 0.00878 | 0.0061 | 0.00609 | 0.00199 | 0.00106 |
|  |  | Velocity of eye movement | degree/s | 0.9 | 0.7 | 0.7 | 0.2 | 0.1 |
|  |  | Ratio of scanpath angles between saccades and horizontal axis | - | 0.00513 | 0.0033 | 0.00343 | 0.0019 | 0.0006 |
|  |  | Fixation count | count | 609 | 206.9 | 194 | 7 | 74.9 |
|  |  | Mean wrap-up gaze duration | ms | 281.8 | 179.9 | 172.4 | 88.5 | 42.8 |
|  |  | First pass mean first fixation duration | ms | 290.2 | 183.1 | 181.6 | 105.9 | 35.7 |
|  |  | Sum of distances between fixations in scanpath | ms | 60147 | 37100.6 | 36000.2 | 14533.5 | 6190.7 |
|  | Language | Variance of acoustic feature (MFCC 2) | - | 307.5 | 159.6 | 157.7 | 38 | 52 |
|  |  | Overall task duration | ms | 196926 | 66222.2 | 57538.5 | 40608 | 26093.7 |
|  |  | Speech rate | count/ms | 0.0056 | 0.0037 | 0.0039 | 0 | 0.00097 |
|  |  | Skewness of acoustic feature (energy) | - | 1.5 | -0.07 | -0.12 | -0.7 | 0.4 |
|  |  | Kurtosis of acoustic feature (MFCC 3) | - | 2.3 | 0.004 | -0.1 | -0.9 | 0.6 |
|  |  | Kurtosis of acoustic feature (MFCC 5) | - | 2.4 | 0.2 | 0.1 | -0.6 | 0.5 |
|  |  | Kurtosis of acoustic feature (MFCC 1) | - | 4.7 | 0.8 | 0.5 | -0.5 | 0.9 |
|  |  | Kurtosis of acoustic feature (MFCC 4) | - | 4.7 | 0.4 | 0.3 | -0.5 | 0.6 |
|  |  | Kurtosis of acoustic feature (MFCC 7) | - | 2.7 | 0.3 | 0.2 | -0.5 | 0.4 |
|  |  | Kurtosis of acoustic feature (MFCC 11 velocity) | - | 1.5 | 0.6 | 0.6 | 0.1 | 0.2 |
| Memory | Language | Skewness of acoustic feature (energy) | - | 1.4 | 0.1 | 0.1 | -0.7 | 0.4 |
|  |  | Variance of acoustic feature (MFCC 2) | - | 278.4 | 148.2 | 141.8 | 58.3 | 49.7 |
|  |  | Kurtosis of acoustic feature (MFCC 5) | - | 2.4 | 0.4 | 0.4 | -0.9 | 0.6 |
|  |  | Variance of acoustic feature (MFCC 8) | - | 215.9 | 135.8 | 133 | 73.2 | 29 |
|  |  | Variance of acoustic feature (MFCC 1) | - | 222.1 | 137 | 135.2 | 44.6 | 39.4 |
|  |  | Kurtosis of acoustic feature (MFCC 2) | - | 2.7 | -0.1 | -0.2 | -0.9 | 0.6 |
|  |  | Variance of acoustic feature (MFCC 5) | - | 350.1 | 163.7 | 161.7 | 83.5 | 43 |
|  |  | Skewness of acoustic feature (MFCC 2) | - | 0.4 | -0.3 | -0.3 | -1.4 | 0.3 |
|  |  | Kurtosis of acoustic feature (MFCC 3) | - | 3.8 | 0.3 | 0.2 | -0.9 | 0.7 |
|  |  | Kurtosis of acoustic feature (MFCC 1) | - | 7.7 | 1.5 | 1.3 | -0.2 | 1.1 |

Supplementary Figure 1: Correlations Between Eye-Movement Features and the Classification Label in the Pupil Calibration Task. Each box in the matrix represents Pearson r correlation between a feature and the classification label. Positive (negative) correlation implies a positive (negative) linear relationship between a feature and a “patient” label. A full list of abbreviations can be found below the figure.


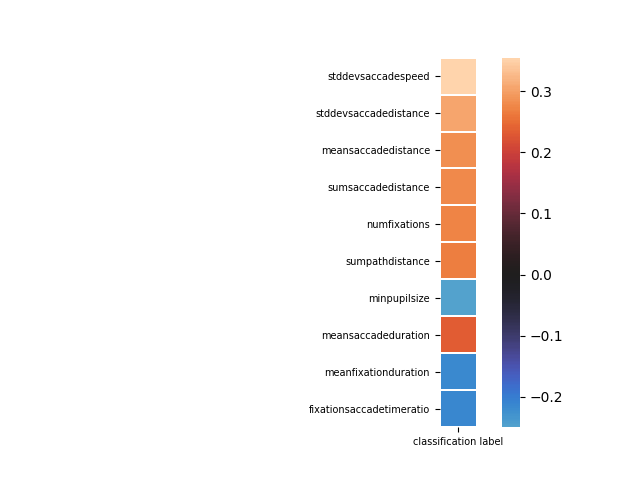


**stddevsaccadespeed**:Standard deviation of saccade speed; **stddevsaccadedistance**: Standard deviation of saccade distance; **meansaccadedistance**: Mean of saccade distance; **sumsaccadedistance**: Sum of saccade distance; **numfixations**: Fixation count; **sumpathdistance**: Sum of path distance; **minpupilsize**:Minimum pupil size; **meansaccadeduration**: Mean of saccade duration; **meanfixationduration**: Mean of fixation duration; **fixationsaccadetimeratio**: Ratio of time spent fixating to saccading

Supplementary Figure 2a: Correlations Between Eye-Movement Features and the Classification Label in the Picture Description Task. Each box in the matrix represents Pearson r correlation between a feature and the classification label. Positive (negative) correlation implies a positive (negative) linear relationship between a feature and a “patient” label. A full list of abbreviations can be found below the figure.


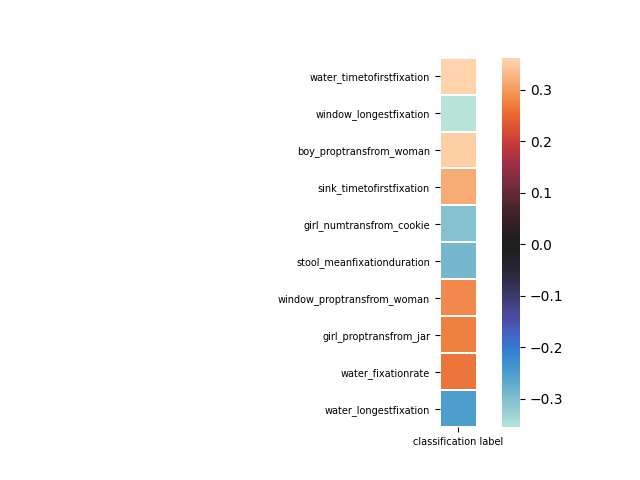


**water_timetofirstfixation**: time before first fixation on “water” Aol; **window_longest_fixation**: longest fixation on “water” Aol; **boy_proptransform_woman**: number of transitions to “boy” Aol from “woman” Aol; **sink_timetofirstfixation**: time before first fixation on “sink” Aol; **girl_numtransfrom_cookie**: number of transitions from “cookie” Aol to “girl” Aol; **stool_meanfixationduration**: mean fixation duration on “stool” Aol; **window_proptransfrom_woman**: number of transitions to “window” Aol from “woman” Aol; **girl_proptransform_woman**: number of transition to “girl” Aol from “jar” Aol; **water_fixationrate**: fixation rate on “water” Aol; **water_longestfixation**: longest fixation on “water” Aol

Supplementary Figure 2b: Correlations Between Language Features and the Classification Label in the Picture Description Task. Each box in the matrix represents Pearson r correlation between a feature and the classification label. Positive (negative) correlation implies a positive (negative) linear relationship between a feature and a “patient” label. A full list of abbreviations can be found below the figure.


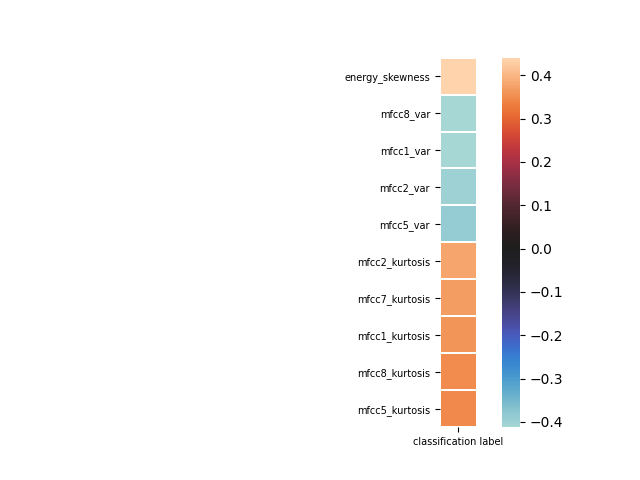


**energy_skewness**: Skewness of acoustic feature (energy); **mfcc8_var**: Variance of acoustic feature (MFCC 8); **mfcc1_var**: Variance of acoustic feature (MFCC 1); **mfcc2_var**: Variance of acoustic feature (MFCC 2); **mfcc5_var**: Variance of acoustic feature (MFCC 5); **mfcc2_kurtosis**: Kurtosis of acoustic feature (MFCC 2); **mfcc7_kurtosis**: Kurtosis of acoustic feature (MFCC 7); **mfcc1_kurtosis**: Kurtosis of acoustic feature (MFCC 1); **mfcc8_kurtosis**: Kurtosis of acoustic feature (MFCC 8); **mfcc5_kurtosis**: Kurtosis of acoustic feature (MFCC 5)

Supplementary Figure 3a: Correlations Between Eye-Movement Features and the Classification Label in the Reading Task. Each box in the matrix represents Pearson r correlation between a feature and the classification label. Positive (negative) correlation implies a positive (negative) linear relationship between a feature and a “patient” label. A full list of abbreviations can be found below the figure.


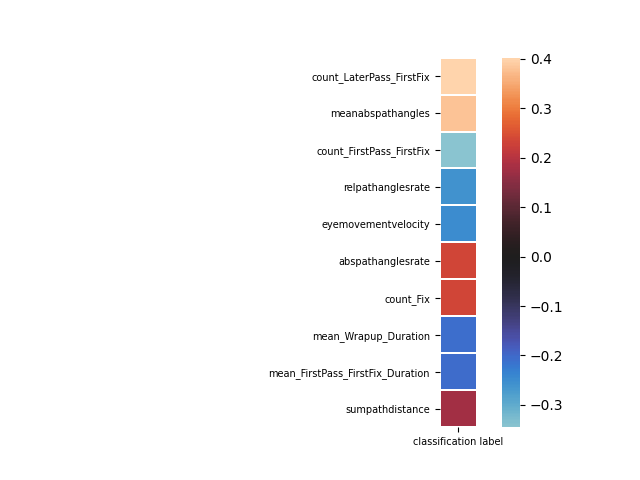


**count_LaterPass_FirstFix**: Later pass first fixation count; **meanabspathangles**: Mean of scanpath angles between saccades and horizontal axis; **count_FirstPass_FirstFix**: First pass first fixation count; **relpathanglesrate**: Ratio of scanpath angles between consecutive saccades; **eyemovementvelocity**: Velocity of eye movement; **abspathanglesrate**: Ratio of scanpath angles between saccades and horizontal axis; **count_Fix**: Fixation count; **mean_Wrapup_Duration**: Mean wrap-up gaze duration; **mean_FirstPass_FirstFix_Duration**: First pass mean first fixation duration; **sumpathdistance**: Sum of distances between fixations in scanpath

Supplementary Figure 3b: Correlations Between Language Features and the Classification Label in the Reading Task. Each box in the matrix represents Pearson r correlation between a feature and the classification label. Positive (negative) correlation implies a positive (negative) linear relationship between a feature and a “patient” label. A full list of abbreviations can be found below the figure.


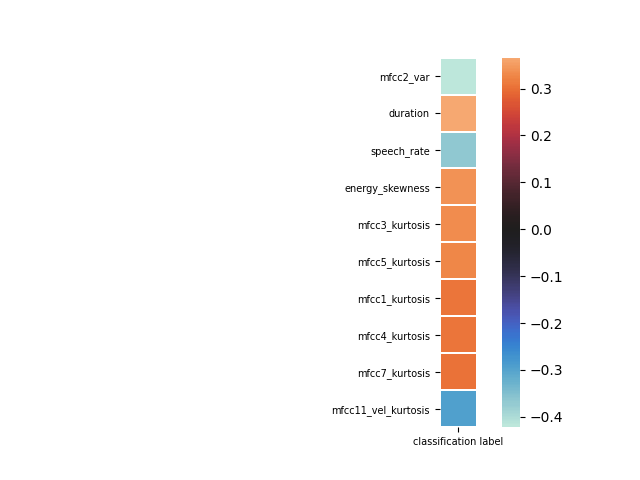


**mfcc2_var**: Variance of acoustic feature (MFCC 2); **duration**: Overall task duration; **speech_rate**: Speech rate; **energy_skewness**: Skewness of acoustic feature (energy); **mfcc3_kurtosis**: Kurtosis of acoustic feature (MFCC 3); **mfcc5_kurtosis**: Kurtosis of acoustic feature (MFCC 5); **mfcc1_kurtosis**: Kurtosis of acoustic feature (MFCC 1); **mfcc4_kurtosis**: Kurtosis of acoustic feature (MFCC 4); **mfcc7_kurtosis**: Kurtosis of acoustic feature (MFCC 7); **mfcc11_vel_kurtosis**: Kurtosis of acoustic feature (MFCC 11 velocity)

Supplementary Figure 4: Correlations Between Language Features and the Classification Label in the Memory Description Task. Each box in the matrix represents Pearson r correlation between a feature and the classification label. Positive (negative) correlation implies a positive (negative) linear relationship between a feature and a “patient” label. A full list of abbreviations can be found below the figure.


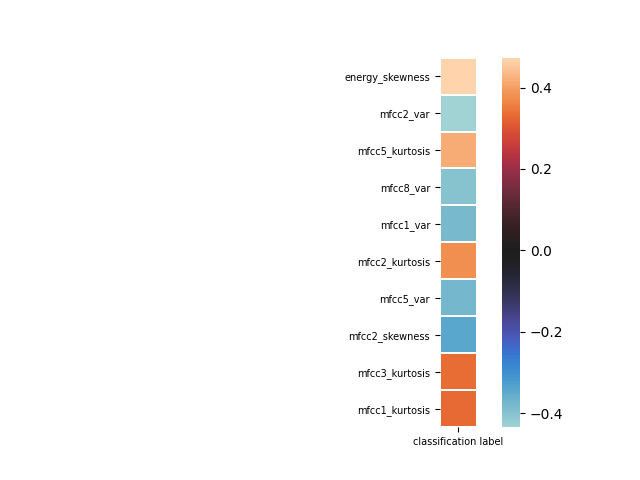


**energy_skewness**: Skewness of acoustic feature (energy); **mfcc2_var**: Variance of acoustic feature (MFCC 2); **mfcc5_kurtosis**: Kurtosis of acoustic feature (MFCC 5); **mfcc8_var**: Variance of acoustic feature (MFCC 8); **mfcc1_var**: Variance of acoustic feature (MFCC 1); **mfcc2_kurtosis**: Kurtosis of acoustic feature (MFCC 2); **mfcc5_var**: Variance of acoustic feature (MFCC 5); **mfcc2_skewness**: Skewness of acoustic feature (MFCC 2); **mfcc3_kurtosis**: Kurtosis of acoustic feature (MFCC 3); **mfcc1_kurtosis**: Kurtosis of acoustic feature (MFCC 1)

##

Supplementary Table 3a. Overall task fusion results with additional performance metrics

| **Model** | **AUC** | **F1 score** | **Accuracy** | **Precision** | **Sensitivity** | **Specificity** |
| --- | --- | --- | --- | --- | --- | --- |
| GNB | 0.82±0.01 | 0.69±0.02 | 0.73±0.01 | **0.77±0.03** | 0.65±0.03 | **0.82±0.02** |
| LR | **0.83±0.01** | **0.72±0.03** | **0.75±0.01** | 0.75±0.04 | **0.72±0.02** | 0.79±0.03 |
| RF | **0.83±0.02** | 0.71±0.03 | 0.74±0.02 | **0.77±0.04** | 0.70±0.03 | 0.80±0.04 |

Supplementary Table 3b. Pupil calibration task model results with additional performance metrics

| **Model** | **AUC** | **F1 score** | **Accuracy** | **Precision** | **Sensitivity** | **Specificity** |
| --- | --- | --- | --- | --- | --- | --- |
| GNB | **0.71±0.02** | **0.64±0.04** | **0.65±0.03** | **0.63±0.02** | **0.72±0.05** | 0.61±0.04 |
| LR | 0.68±0.02 | 0.58±0.03 | 0.63±0.02 | **0.63±0.02** | 0.60±0.03 | 0.68±0.03 |
| RF | 0.63±0.05 | 0.50±0.05 | 0.59±0.04 | 0.59±0.06 | 0.49±0.06 | **0.70±0.04** |

Supplementary Table 3c. Picture description task model results with additional performance metrics

| **Model** | **AUC** | **F1 score** | **Accuracy** | **Precision** | **Sensitivity** | **Specificity** |
| --- | --- | --- | --- | --- | --- | --- |
| GNB | **0.80±0.02** | **0.68±0.03** | **0.73±0.02** | **0.75±0.04** | 0.65±0.03 | **0.80±0.02** |
| LR | 0.79±0.01 | 0.67±0.02 | 0.70±0.02 | 0.69±0.03 | **0.69±0.03** | 0.71±0.04 |
| RF | 0.77±0.02 | 0.64±0.02 | 0.68±0.02 | 0.71±0.04 | 0.62±0.03 | 0.76±0.04 |

Supplementary Table 3d. Reading task model results with additional performance metrics

| **Model** | **AUC** | **F1 score** | **Accuracy** | **Precision** | **Sensitivity** | **Specificity** |
| --- | --- | --- | --- | --- | --- | --- |
| GNB | 0.78±0.01 | 0.55±0.02 | 0.67±0.01 | **0.77±0.03** | 0.46±0.03 | **0.87±0.02** |
| LR | 0.80±0.01 | 0.67±0.03 | 0.71±0.02 | 0.74±0.04 | 0.65±0.03 | 0.78±0.03 |
| RF | **0.82±0.02** | **0.7±0.03** | **0.74±0.03** | **0.77±0.04** | **0.68±0.03** | 0.8±0.04 |

Supplementary Table 3e. Memory description task model results with additional performance metrics

| **Model** | **AUC** | **F1 score** | **Accuracy** | **Precision** | **Sensitivity** | **Specificity** |
| --- | --- | --- | --- | --- | --- | --- |
| GNB | **0.78±0.01** | **0.64±0.02** | **0.69±0.01** | **0.72±0.02** | 0.6±0.02 | **0.78±0.01** |
| LR | 0.72±0.02 | 0.63±0.02 | 0.65±0.02 | 0.65±0.03 | **0.64±0.03** | 0.66±0.03 |
| RF | 0.72±0.04 | 0.6±0.02 | 0.65±0.02 | 0.69±0.04 | 0.57±0.03 | 0.74±0.04 |

Supplementary Table 4. Details and descriptive statistics about the top 5 most important LR features, based on feature importance analysis. All features are continuous. In the unit column, “-” signifies that the unit is not human intuitive. A positive (+) direction (odds ratio > 1) represents a higher feature value in the patient group. A negative (–) direction represents a higher feature value for the control group. *: *p* < *0.05*

| **Task** | **Modality** | **Feature** | **+/-** | **Odds ratio** | **95% CI** | **Units** | **Max** | **Mean** | **Median** | **Min** | **Standard deviation** |
| --- | --- | --- | --- | --- | --- | --- | --- | --- | --- | --- | --- |
| Pupil | Eye movement | Standard deviation of saccade speed | + | 1.46 | (0.63, 3.37) | degree/s | 15 | 1.2 | 0.9 | 0 | 1.4 |
|  |  | Mean fixation duration | – | 0.9997 | (0.9989, 1.0005) | ms | 8274 | 855.2 | 469.6 | 101 | 1077.9 |
|  |  | Standard deviation of saccade distance | + | 1 | (0.99, 1.02) | degrees | 361.7 | 75 | 56.8 | 0 | 69.8 |
|  |  | Ratio of time spent fixating to saccading | – | 1 | (0.98, 1.01) | _ | 367.1 | 55.6 | 38.8 | 3.5 | 53.1 |
|  |  | Sum of saccade distance | + | 1.0003 | (0.9988, 1.0019) | degrees | 5834.5 | 841.8 | 632.8 | 33.1 | 807.9 |
| Picture Description | Eye movement | Longest fixation on window AoI | – | 0.998 | (0.996, 1.000) | ms | 2391 | 649.9 | 571 | 175 | 341.  4 |
|  |  | Number of transitions from curtain AoI to window AoI | – | 0.83 | (0.66, 1.07) | count | 0.062 | 0.015 | 0.013 | 0 | 0.014 |
|  |  | Number of transitions from boy AoI to cookie AoI | – | 0.4 | (0.10, 1.70) | count | 0.25 | 0.021 | 0 | 0 | 0.047 |
|  |  | Time before first fixation on water AoI | + | 1.00006 | (0.99996, 1.0015) | ms | 59877 | 11786.7 | 9961 | 1367 | 10378.5 |
|  |  | Number of transitions from dishcloth AoI to window AoI | + | 2.57 | (0.46, 14.52) | count | 0.12 | 0.0024 | 0 | 0 | 0.047 |
|  | Language | Mentions of exterior information unit | – | 0.43* | (0.18, 0.99) | count | 14 | 1.4 | 1 | 0 | 1.8 |
|  |  | Variance of acoustic feature (MFCC 8) | – | 0.97 | (0.93, 1.00) | - | 189.8 | 120.5 | 120.6 | 61.6 | 28.8 |
|  |  | Distribution of acoustic feature (MFCC 6) | – | 0.36 | (0.11, 1.15) | - | 7 | 0.7 | 0.5 | -0.8 | 1.1 |
|  |  | Mean of acoustic feature (MFCC 5) | + | 1.17 | (0.97, 1.41) | - | 11.8 | -5.4 | -5.6 | -16.5 | 4.5 |
|  |  | Mean of acoustic feature (MFCC 4) | + | 1.15 | (0.95, 1.39) | - | 6.2 | -6.7 | -6.7 | -19.8 | 4.3 |
| Reading | Eye  movement | Refixation count | + | 1.03 | (0.99, 1.09) | count | 429 | 58.1 | 40 | 1 | 61 |
|  |  | Later pass first fixation count | + | 1.04 | (0.97, 1.11) | count | 131 | 36.6 | 32 | 2 | 22 |
|  |  | Fixation count | – | 0.66 | (0.25, 1.79) | count | 609 | 206.9 | 194 | 7 | 74.9 |
|  |  | Mean saccade distance | – | 0.99 | (0.98, 1.01) | degrees | 7.9 | 2.3 | 2 | 0.7 | 1.3 |
|  |  | Mean wrap-up gaze duration | – | 0.99 | (0.96, 1.02) | ms | 252.5 | 93.8 | 89.6 | 20 | 38.4 |
|  | Language | Variance of acoustic feature (MFCC 12) | – | 0.95* | (0.92, 0.99) | - | 131.2 | 78.3 | 76.7 | 48.5 | 16.1 |
|  |  | Distribution of acoustic feature (energy acceleration) | + | 1.17 | (0.99, 1.38) | - | 55.6 | 16.8 | 15.5 | 6.6 | 6.3 |
|  |  | Mean of acoustic feature (MFCC 3) | + | 1.12 | (0.99, 1.26) | - | 22 | 1.7 | 0 | -8.8 | 6.7 |
|  |  | Variance of acoustic feature (MFCC 2) | – | 0.99 | (0.98, 1.00) | - | 307.5 | 159.6 | 157.7 | 38 | 52 |
|  |  | Overall task duration | + | 1.00004 | (0.99999, 1.00010) | ms | 196926 | 66222.2 | 57538.5 | 40608 | 26093.  7 |
| Memory | Language | Variance of acoustic feature (MFCC 8) | – | 0.98* | (0.95, 1.00) | - | 215.9 | 135.8 | 133 | 73.2 | 29 |
|  |  | Mean of acoustic feature (MFCC 3) | – | 0.99 | (0.97, 1.00) | - | 23.8 | 2.4 | 0.7 | -8.7 | 6.5 |
|  |  | Variance of acoustic feature (MFCC 2) | + | 1.47 | (0.80, 2.72) | - | 278.4 | 148.2 | 141.8 | 58.3 | 49.7 |
|  |  | Mean of acoustic feature (energy) | + | 1.45 | (0.75, 2.78) | - | 16.9 | 14.6 | 14.6 | 11.8 | 1 |
|  |  | Distribution of acoustic feature (MFCC 4) | + | 1.01 | (0.99, 1.02) | - | 4.6 | 0.8 | 0.6 | -0.5 | 0.8 |
